# Immune-Stimulatory and Therapeutic Activity of *Tinospora cordifolia*: Double-Edged Sword against Salmonellosis

**DOI:** 10.1155/2017/1787803

**Published:** 2017-11-26

**Authors:** Sultan Alsuhaibani, Masood A. Khan

**Affiliations:** ^1^Department of Medical Laboratory, College of Applied Medical Sciences, Qassim University, Buraydah, Saudi Arabia; ^2^Department of Optometry, College of Applied Medical Sciences, Qassim University, Buraydah, Saudi Arabia

## Abstract

The present study was aimed at determining the activity of aqueous and methanolic extracts of *Tinospora cordifolia* (AETC and METC) against *Salmonella typhimurium*. *In vitro* anti-*Salmonella* activity of *T. cordifolia* was determined through the broth dilution and agar well diffusion assays. The immune-stimulating potential of AETC or METC was determined by measuring the cytokine levels in the culture supernatants of treated murine J774 macrophages. Antibacterial activity of AETC or METC was determined by treating *S. typhimurium-*infected macrophages and BALB/C mice. The toxicity of AETC or METC was determined by measuring the levels of liver inflammation markers aspartate transaminase (AST) and alanine transaminase (ALT) and antioxidant enzymes. Macrophages treated with AETC or METC secreted greater levels of IFN-*γ*, TNF-*α*, and IL-1*β*. METC showed greater activity against *S. typhimurium* infection in macrophages and mice as well. Treatment with METC resulted in increased survival and reduced bacterial load in *S. typhimurium*-infected mice. Moreover, METC or AETC treatment reduced the liver inflammation and rescued the levels of antioxidant enzymes in *S. typhimurium*-infected mice. The results of the present study suggest that the use of *T. cordifolia* may act as a double-edged sword in combating salmonellosis.

## 1. Introduction

Antibiotics in a modern therapeutic system have been tremendously used in controlling the infectious diseases [1]. Due to the extensive use of antibiotics, there has been an emergence of multidrug-resistant strains of many pathogens that are posing serious challenges to the clinicians [2]. There is a dire need to find suitable replacements for some of the currently used antibiotics [3–5]. Moreover, some antibacterial and antifungal agents exhibit serious untoward effects in the treated persons [6–8].

*Salmonella* infections pose an important public health problem all over the world [9]. *Salmonella* spp. cause a variety of diseases, from enteritis to fatal infections in animals, and food-borne infection to typhoid fever in humans. Typhoid is one of the most communicable diseases in India [4]. Recent reports of decreased susceptibility of *Salmonella* to some antibiotics are a matter of big concern among the clinicians and scientists [4]. The intracellular survival of *Salmonella* hinders its elimination from the host and thus the treatment of typhoid. Moreover, *Salmonella* adopts many strategies to evade the immune system of the host [10, 11].

*T. cordifolia*, commonly known as Guduchi or Giloy, is used as a medicine for centuries in the Ayurvedic and Unani systems of the medicine. *T. cordifolia* extract contains many constituents such as alkaloids, steroids, glycosides, and polysaccharides [12]. It has been shown to possess antidiabetic, antioxidant, antihepatotoxic, and immunomodulatory properties [13, 14]. The aqueous extract of *T. cordifolia* has been shown to protect against *Escherichia coli* and *Staphylococcus aureus* infections [15, 16]. *T. cordifolia* enhances the phagocytic and intracellular bactericidal activities of macrophages and neutrophils against *E. coli*-induced peritonitis [15]. The active ingredient, G 1-4A, of a dry stem of *T. cordifolia* protected mice against lipopolysaccharide- (LPS-) induced endotoxic shock by modulating the responses of macrophages [17]. It has been shown to control the drug-resistance *Mycobacterium tuberculosis* infection by inducing Th1 immune responses [18]. *T. cordifolia* extract showed an antitumor potential against the skin carcinogenesis in a mouse model [19].

In the present study, we determined the activity of the aqueous and methanolic extracts of *T. cordifolia* against *S. typhimurium*. The results showed that *T. cordifolia* was effective in controlling *S. typhimurium* growth in macrophages, as well as in mice.

## 2. Materials and Methods

### 2.1. Materials

Nutrient Broth was purchased from Hi Media Pvt. Ltd. Mumbai, India. *S. typhimurium* (ATCC number 23564) was obtained from the American Type Culture Collection (ATCC), Rockville, USA. High-performance liquid chromatography (HPLC) grade methanol was purchased from Thermo-Fisher Scientific (Waltham, MA, USA). Cytokines IFN-*γ*, TNF-*α*, and IL-1*β* and ELISA kits were purchased from PeproTech (Rocky Hill, NJ, USA). Superoxide dismutase (SOD) and catalase (CAT) estimation kits were purchased from Biovision Inc. (Milpitas, CA, USA). The kit of the liver inflammation markers, ALT and AST, was bought from Quimica Clinica Aplicada (Amposta, Tarragona, Spain).

### 2.2. Mice

BALB/C mice at 12 weeks of age (weighing 24 ± 4 g each) were obtained from the animal house facility of the College of Applied Medical Sciences, Qassim University. The techniques used for bleeding, injection, and sacrifice of animals were approved by the animal ethics committee of the college.

### 2.3. Macrophage Cell Line

The murine macrophage cell line J 774 was maintained in Dulbecco's modified Eagle's medium (DMEM) as described earlier [20].

### 2.4. Preparation of Extracts from the Stems of *T. cordifolia*

The dried stems of *T. cordifolia* were procured from the herbal store of Hakeem Ajmal Khan Unani Tibbiya College, Aligarh Muslim University, Aligarh, India. The stems were powdered, and aqueous and methanolic extract of *T. cordifolia* (AETC and METC) was prepared as described previously [21]. Briefly, 25 grams of powder was soaked in 250 ml of methanol for 12 hours with continuous stirring. The suspensions were refluxed under the reduced pressure for 6 hours and filtered through the Whatman filter paper (number 1). The filtrates containing methanol were concentrated using a rotary evaporator, whereas aqueous extracts were dried using a water bath.

### 2.5. Determination of Antibacterial Activity of *T. cordifolia* Extract

The anti-*Salmonella* activity of AETC or METC was carried out by agar well diffusion method [21]. *S. typhimurium* culture was swabbed over nutrient agar plates using a sterile cotton swab and wells were made using sterile well cutter (6 mm). Various concentrations (25, 50, and 100 *μ*g/well) of AETC or METC were aseptically transferred to the wells and incubated at 37°C. After 24 hours, the diameter of the zone of inhibition was measured.

### 2.6. Minimum Inhibitory Concentration (MIC) of *T. cordifolia* Extract

Agar diffusion method is a qualitative method useful for the detection of the antimicrobial properties. But it is not the correct method to determine the relative antimicrobial activity because less active and more diffusible extract can show an increased zone of inhibition compared to more active and less diffusible extract. Thus, it is important to determine the minimum inhibitory concentrations (MICs) of the extract in the solution. MIC was determined by using the broth dilution method [22]. Dried AETC and METC were weighed and suspended in water to make 20 mg/ml concentration. A range of concentration of AETC or METC (1 *μ*g/ml to 2000 *μ*g/ml) was taken on a 96-well microtiter plate containing broth medium. Thereafter, 100 *μ*l of inoculum containing 1 × 10^5^ CFU of *S. typhimurium* was added to each well. Wells containing *S. typhimurium* (no drug or extract) were used as a negative control. The concentrations (0.01–100 *μ*g/ml) of standard antibacterial drug cefixime were also used as a positive control. The microtiter plates were incubated for 24 hours at 37°C. The lowest concentration of the extract that showed no visible growth after incubation was considered the MIC value of the extract.

### 2.7. Effect of the Treatment of AETC or METC on Cytokine Production

Murine macrophage cell lines (J774) were seeded into 24-well cell culture plates at a density of 2 × 10^5^ cells per well and incubated at 37°C for 24 hours. Cells were washed and treated with various doses (0, 100, 200, and 500 *μ*g/ml) of AETC or METC. After 24 hours of treatment, the supernatants were collected and the amounts of interferon-gamma (IFN-*γ*), tumor necrosis factor-alpha (TNF-*α*), and interleukin-1 beta (IL-1*β*) were determined by ELISA as described earlier [23].

### 2.8. Effect of AETC and METC on the Intracellular Multiplication of *S. typhimurium*

The anti-*Salmonella* activity of AETC or METC was determined against the intracellular growth of *S. typhimurium* in macrophages as described earlier [24]. Macrophages were seeded in triplicates in 24-well, flat-bottomed sterile culture plates with 1 × 10^5^ cells/well in DMEM with 10% heat-inactivated fetal bovine serum (FBS) and incubated at 37°C in 5% CO_2_ for 24 hours. Cells were washed and fresh DMEM was added. Each well was infected with *S. typhimurium* (5 × 10^5^ CFU/well) in a minimum volume of DMEM. After 4 hours of incubation, cells were washed to remove unphagocytosed bacteria. Various concentrations of AETC or METC were added to each well as described in the above section. After 24 h of incubation, macrophages were lysed with 0.1% Tween-20 and bacteria were recovered after centrifugation. The number of colony-forming units (CFU) of *S. typhimurium* was determined by culturing on Luria agar media after incubation at 37°C for 24 hours.

### 2.9. Infection of BALB/C Mice with *S. typhimurium*

*S. typhimurium* cells were washed with sterile normal saline at low-speed centrifugation (2000 rpm) and diluted to the appropriate concentrations in saline just prior to injecting. Each mouse was infected intravenously with a lethal dose of 5 × 10^5^ viable *S. typhimurium* bacteria as described earlier [21].

### 2.10. Treatment of *S. typhimurium-*Infected Mice with *T. cordifolia*

Mice were treated at two different doses (50 and 100 mg/kg) of AETC or METC orally for 7 days (day 1 to day 7) after *S. typhimurium* infection (day 0). Standard antibiotic cefixime was used at a dose of 5 mg/kg.

Mice were divided into following groups: (1) saline, (2) AETC-50, (3) AETC-100, (4) METC-50, (5) METC-100, and (6) cefixime-5 mg/kg, and each group contained 10 mice. The mice were observed daily for their morbidity and mortality.

### 2.11. Quantitative Analysis of *S. typhimurium* in the Spleen

The efficacy of the treatment was determined by assessing the survival and bacterial load in the spleen of mice untreated or treated with *T. cordifolia* extract. Three mice from each group were sacrificed on day 5 post *S. typhimurium* infection, and the spleen was taken out aseptically as described earlier [21, 22]. Equally weighed portions of the spleen tissues were homogenized in 5 ml of sterile normal saline, and various dilutions of the suspension were plated on NB agar plates. The plates were incubated at 37°C for 24–36 hours. The number of viable *S*. *typhimurium* colonies was counted and the bacterial load was determined by multiplying by the dilution factor.

### 2.12. Biochemical Analysis

On day 5 posttreatment, the levels of antioxidant enzymes such as superoxide dismutase (SOD) and catalase (CAT) were determined in the spleen tissue homogenates as described earlier [25]. The spleen tissues from untreated or treated groups of mice were rinsed in cold phosphate-buffered saline (PBS) and the connective tissue was removed. The tissue samples were then homogenized with PBS and centrifuged at 5000*g* for 15 min at 4°C to collect the supernatant fractions, which were used to assay SOD and CAT activities.

To determine the liver toxicity, the levels of aspartate transaminase (AST) and alanine transaminase (ALT), the markers of liver inflammation, were determined in the blood of untreated or treated groups of mice [25].

### 2.13. Statistical Analysis

Analysis of the survival of mice was performed using Kaplan–Meier curve, and various groups were compared by log-rank test. Bacterial burden (CFU) in the spleen was analyzed by one-way ANOVA using GraphPad Prism software version 5.0.

## 3. Results

### 3.1. AETC and METC Showed *In Vitro* Anti-*Salmonella* Activity

AETC or METC showed potent activity against the present strain of *S. typhimurium* as measured by the zone of inhibition. The anti-*Salmonella* activity of METC was higher as compared to that of AETC. The zone of inhibition was found to be 4, 6, and 12 mm in wells containing 25, 50, and 100 *μ*g of METC, respectively, whereas there were 1, 3, and 5 mm of zones of inhibition in wells containing the same quantities of AETC.

Minimum inhibitory concentration (MIC) of AETC or METC was determined against *S. typhimurium* by seeing the turbidity of the growth medium. *S. typhimurium* did not show any visible growth at a concentration of 32 *μ*g/ml of METC or 64 *μ*g/ml of AETC. Whereas the standard drug cefixime showed MIC at a concentration of 0.20 *μ*g/ml.

### 3.2. Treatment with AETC or METC Stimulated the Secretion of Proinflammatory Cytokines by Macrophages

**Figure 1:**
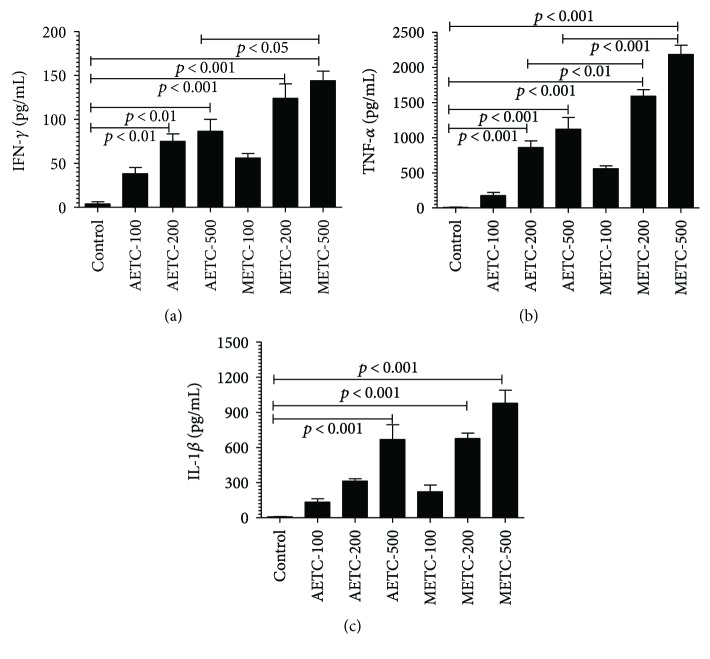
Secretion of cytokines by macrophages treated with AETC or METC. Murine macrophage J774 cells were treated at the doses of 100, 200, and 500 *μ*g/ml of AETC or METC for 24 hours at 37°C. The levels of cytokines (a) IFN-*γ*, (b) TNF-*α*, and (c) IL-1*β* were determined in the supernatants by ELISA.

The effect of AETC or METC on the production of cytokines by macrophages was assessed by determining the levels of proinflammatory cytokines, including IFN-*γ*, TNF-*α*, and IL-1*β* in the culture supernatants of untreated or treated macrophages. The levels of IFN-*γ*, TNF-*α*, and IL-1*β* were higher in the supernatant of macrophages treated either with AETC or METC when compared to untreated macrophages (Figure 1(a)). Macrophages treated with AETC at the doses of 100, 200, and 500 *μ*g/ml produced 38.33 ± 6.888, 75.00 ± 8.660, and 86.67 ± 13.48 pg/ml of IFN-*γ*, respectively, whereas the macrophages treated with similar doses of METC produced 56.00 ± 5.292, 124.0 ± 16.65, and 144.0 ± 11.02 pg/ml of IFN-*γ* (Figure 1(a)). Macrophages in the control group secreted almost undetectable amounts (0–9 pg/ml) of IFN-*γ* (Figure 1(a)).

The level of TNF-*α*, an important proinflammatory cytokine, was also measured in the supernatants of macrophages untreated or treated with AETC or METC. Macrophages treated with AETC at the doses of 100, 200, and 500 *μ*g/ml produced 175.3 ± 44.46, 859.0 ± 93.63, and 1123 ± 164.1 pg/ml of TNF-*α*, respectively, whereas the macrophages treated with similar doses of METC produced 559.7 ± 38.77, 1591 ± 94.03, and 2185 ± 131.1 pg/ml of TNF-*α* (Figure 1(b)). The secretion of TNF-*α* was undetectable in the supernatant of control group of macrophages (Figure 1(b)).

Like IFN-*γ* and TNF-*α*, the level of IL-1*β* was also significantly higher in the supernatants of macrophages treated with AETC or METC. Macrophages treated with AETC at the doses of 100, 200, and 500 *μ*g/ml produced 132.7 ± 29.36, 313.3 ± 19.72, and 667.7 ± 127.1 pg/ml of IL-1*β*, whereas treatment with similar doses of METC resulted in the production of 222.3 ± 56.91, 676.3 ± 47.01, and 978.3 ± 111.6 pg/ml of IL-1*β* (Figure 1(c)). Macrophages in the control group secreted IL-1*β* in the range of 4–12 pg/ml (Figure 1(c)).

### 3.3. Treatment with AETC or METC Inhibited the Intracellular Multiplication of *S. typhimurium*

**Figure 2:**
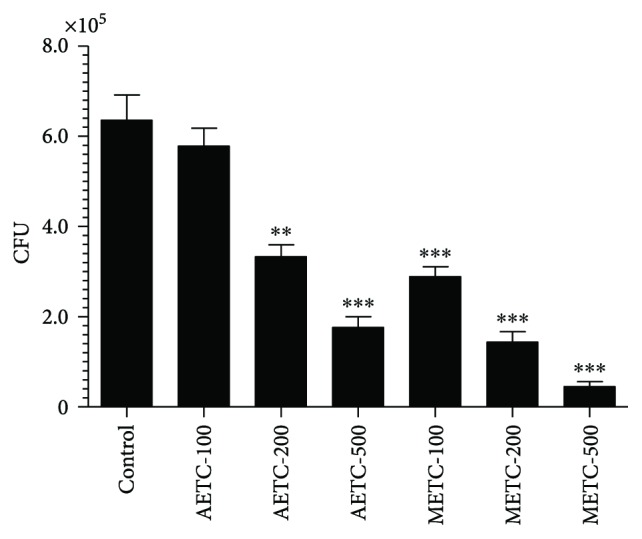
Treatment with AETC or METC inhibits the intracellular survival of *S. typhimurium* in macrophages. Macrophages were infected with *S. typhimurium* at a MOI = 5. After 4 hours of incubation, cells were washed to remove unphagocytosed bacteria. Cells were treated with various concentrations (100, 200, and 500 *μ*g/ml). After 24 h of incubation, macrophages were lysed with 0.1% Tween-20 and bacteria were recovered after centrifugation. CFUs of *S. typhimurium* were determined by culturing on Luria agar media at 37°C for 24 hours. Results shown are representative of three independent experiments and presented here as mean ± SD. ^∗∗^*p* < 0.01 and ^∗∗∗^*p* < 0.001 as compared to untreated infected control.

The effect of AETC or METC on the intracellular survival of *S. typhimurium* was assessed by treating the infected macrophages. Treatment with AETC or METC substantially decreased the bacterial burden in the treated macrophages. There was a greater reduction in bacterial load in macrophages treated with METC as compared to that in AETC-treated macrophages (Figure 2). The bacterial load in the infected macrophages, neither treated with AETC nor METC, was found to be 635475 ± 96803. The colony forming units (CFUs) of *S. typhimurium* in macrophages treated with METC at the doses of 100, 200, and 500 *μ*g/ml were found to be 2.9 × 10^5^, 1.44 × 10^5^, 4.5 × 10^4^, respectively, as compared to 5.78 × 10^5^, 3.32 × 10^5^, and 1.76 × 10^5^ CFUs in macrophages treated with the same doses of AETC, respectively (Figure 2).

### 3.4. Administration of AETC or METC Increased the Survival of *S. typhimurium-*Infected Mice

**Figure 3:**
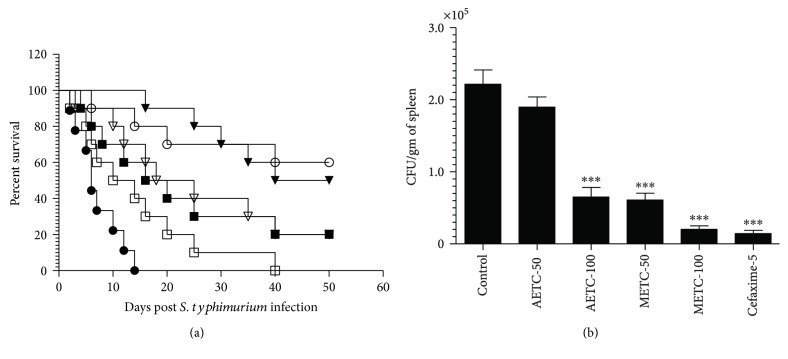
Treatment with AETC or METC increases the survival of S*. typhimurium*-infected mice and reduces bacterial load in the spleen of infected mice. (a) Mice were infected with 5 × 10^5^ CFU of *S. typhimurium* through intravenous route. After 24 hours of infection, mice were orally treated with AETC or METC at the doses of 50 and 100 mg/kg for consecutive 7 days postinfection. A group of mice was treated with standard antibiotic cefixime at the dose of 5 mg/kg. Mice were observed for 50 days to check their survival. Untreated control (●), AETC-50 mg/kg (□), METC-50 mg/kg (▽), AETC-100 mg/kg (■), METC-100 mg/kg (▼), and cefixime-5 mg/kg (○). Untreated control versus AETC-100 mg/kg (*p* < 0.01), untreated control versus METC-100 mg/kg (*p* < 0.001), untreated control versus cefixime-5 mg/kg (*p* < 0.001). (b) On day 5 post *S. typhimurium* infection, three mice from untreated or treated groups were sacrificed and their spleen was taken out for homogenization. The spleen tissue homogenates were cultured to determine the bacterial load as described in the method section. ^∗∗∗^*p* < 0.001 as compared to untreated control.

Therapeutic effect of AETC or METC was determined by treating *S. typhimurium*-infected mice at the doses of 50 and 100 mg/kg for 7 consecutive days. Mice were observed for 50 days to monitor the survival. The mortality rate in the untreated group of mice was found to be 100% by day 15 postinfection, whereas all mice in the group treated at a dose of 50 mg/kg of AETC died by day 40 postinfection (Figure 3(a)). However, the mice in the group treated at a dose of 100 mg/kg of AETC showed 20% survival rate on day 50 postinfection (Figure 3(a)). The median survival of mice in the untreated group was found to be 7 days, whereas mice in the groups treated with AETC at the doses of 50 mg/kg and 100 mg/kg had a median survival of 12 and 18 days, respectively. The survival rate of mice in the group treated with AETC at a dose of 100 mg/kg was found to be significantly greater as compared to that of mice in the untreated group (*p* < 0.01).

On the other hand, the mice in the group treated at the doses of 50 and 100 mg/kg of METC showed 20% and 50% survival rate, respectively. The median survival of mice in the groups treated with METC at the doses of 50 mg/kg and 100 mg/kg was found to be 21.5 and 45 days, respectively, which were significantly greater to that (6 days) of the untreated group of mice (*p* < 0.001). The mice in the group treated with cefixime at a dose of 5 mg/kg showed 60% survival rate with a median survival of >45 days (Figure 3(a)).

The severity of *S. typhimurium* infection was determined by culturing the spleen tissue homogenates from untreated or treated mice on the NB agar plates. There were the highest numbers of CFUs (221688 ± 34067) in the spleen tissue homogenates of untreated mice (Figure 3(b)). Mice in the groups treated at the doses of 50 and 100 mg/kg of AETC showed a lower bacterial load (189892 ± 24147 and 65057 ± 23096, resp.) in their spleen (Figure 3(b)). Treatment with AETC at a dose of 100 mg/kg showed a significant reduction in the bacterial load as compared to the mice in the untreated group (*p* < 0.001). Moreover, the mice in the groups treated at the doses of 50 and 100 mg/kg of METC showed greater reduction in the bacterial load (60876 ± 16656 and 20357 ± 8156, resp.) (Figure 3(b)) and this reduction was found to be significant when compared to untreated mice (*p* < 0.001). These results suggested that METC showed anti-*Salmonella* activity superior to AETC at the same dose. However, the treatment with cefixime (5 mg/kg) resulted in the highest reduction in the bacterial load (14455 ± 7864) as compared to all other groups in the study (Figure 3(b)).

### 3.5. Treatment with AETC or METC Reduces the Liver Inflammation in *S. typhimurium*-Infected Mice

**Figure 4:**
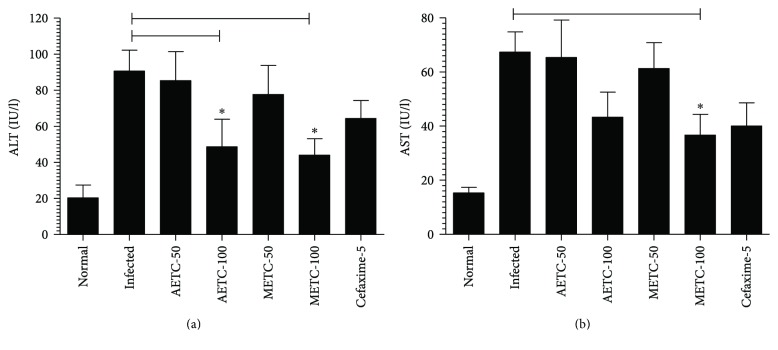
The levels of ALT and AST in the sera of *Salmonella*-infected mice untreated or treated with AETC or METC. Blood was drawn from the mice of various groups on day 5 posttreatment. The levels of (a) ALT and (b) AST were determined in the sera of mice. Results shown are representative of three independent experiments and presented here as mean ± SD. ^∗^*p* < 0.05 as compared to untreated infected mice.

The levels of AST and ALT were measured in the serum samples of *Salmonella*-infected mice untreated or treated with AETC or METC. *Salmonella*-infected mice showed the ALT level of 90.67 ± 11.62, which was significantly higher to an ALT level of 20.33 ± 4.096 in normal mice (Figure 4(a)) (*p* < 0.05). The level of ALT in *Salmonella*-infected mice was significantly reduced from 90.67 ± 11.62 to 48.67 ± 8.819 in AETC-treated mice and from 90.67 ± 11.62 to 44.00 ± 5.29 in METC-treated mice at a dose of 100 mg/kg (*p* < 0.05).

Like the ALT level, the level of AST was also significantly increased in *Salmonella*-infected mice (67.33 ± 7.513) as compared to normal mice (15.33 ± 2.028) (*p* < 0.05) (Figure 4(b)). Treatment with AETC at a dose of 100 mg/kg reduced the levels of AST from 67.33 ± 7.513 to 43.33 ± 9.262 (Figure 4(b)). More importantly, treatment with METC at the same dose significantly reduced the level of AST from 67.33 ± 7.513 to 36.67 ± 7.688 (*p* < 0.05).

### 3.6. Treatment with AETC or METC Compensates the Depleted Levels of Antioxidant Enzymes in *Salmonella*-Infected Mice

**Figure 5:**
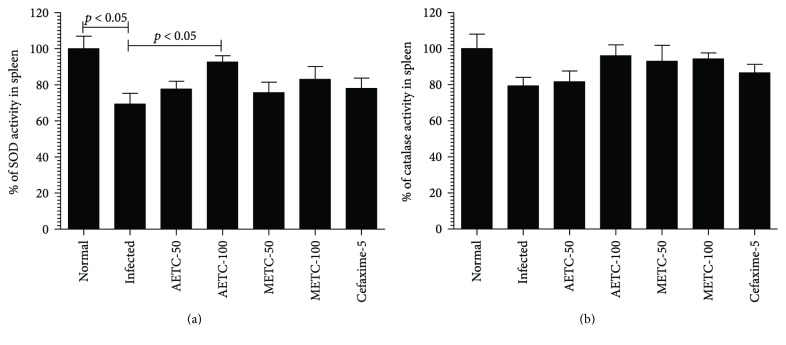
The levels of SOD and CAT in the spleen tissue homogenates of *Salmonella*-infected mice untreated or treated with AETC or METC. The spleen was taken from the mice of various groups on day 5 posttreatment. The spleen tissues were rinsed in cold phosphate-buffered saline (PBS) and homogenized in PBS followed by centrifugation at 5000*g* to collect the supernatant. The activities of (a) SOD and (b) CAT were determined in the supernatants. Results shown are representative of three independent experiments and presented here as mean ± SD. *p* < 0.05 as compared to untreated infected mice.

The levels of antioxidant enzymes such as SOD and CAT were measured in the spleen tissue homogenate of normal or *Salmonella*-infected mice untreated or treated with AETC or METC.  Figure 5(a) shows that SOD level in the spleen homogenate of *Salmonella-*infected mice was about 70% of uninfected normal mice that was significantly reduced (*p* < 0.05). Treatment with AETC and METC reversed the depleted level of SOD in *Salmonella*-infected mice. This effect was found to be significant (from 70% to 92%) in the group of mice treated with AETC at a dose of 100 mg/kg (*p* < 0.05). Although the treatment with METC increased SOD level from 70% to 83%, cefixime treatment resulted in an increase of SOD from 70% to 78%, which was statistically insignificant (Figure 5(a)).

Similar to SOD, the level of catalase was also found to be reduced in *Salmonella*-infected mice (Figure 5(b)). Although the CAT level was not significantly decreased in *Salmonella*-infected mice as compared to uninfected mice, treatment with AETC or METC or cefixime also reversed the depleted levels of CAT in *Salmonella*-infected group of mice (Figure 5(b)).

### 3.7. *T. cordifolia* Treatment Polarizes the Macrophages

Macrophages treated with AETC or METC secreted higher amounts of IFN-*γ*, TNF-*α*, and IL-1*β* that are characteristics of classically activated macrophages (M1). This shows that treatment with *T. cordifolia* extract polarizes the macrophages in favour of M1 type.

## 4. Discussion

Plants and their derived extracts have been used for many hundreds of years in pharmaceuticals as the alternative medicines and natural therapies. Plant extracts are potential sources of novel antimicrobial compounds, especially against bacterial pathogens [26]. The emergence of microbial resistance to many presently available antibiotics has resulted in morbidity and mortality from treatment failure and increased health care costs [27, 28]. There is a dire need to find for new, safe, and effective bioactive agents that can fight the problem of multidrug resistance. In the present work, we showed the efficacy of *T. cordifolia* extract against *S. typhimurium* both *in vitro* and *in vivo* studies.

The stem of *T. cordifolia* has been used as a constituent in many Ayurvedic and Unani preparations for the treatment of general debility, dyspepsia, fever, and urinary diseases [29]. The stem is used as diuretic, stimulates bile secretion, and cures jaundice [30]. The extract of the stem is also useful in skin diseases and in combination with other drugs act as an antidote to snakebite [31]. The dry bark of *T. cordifolia* has been shown to possess antipyretic, antiallergic, anti-inflammatory, and antileprotic properties [32–35]. Moreover, *T. cordifolia* has been shown to be effective against diabetes mellitus [36].

**Figure 6:**
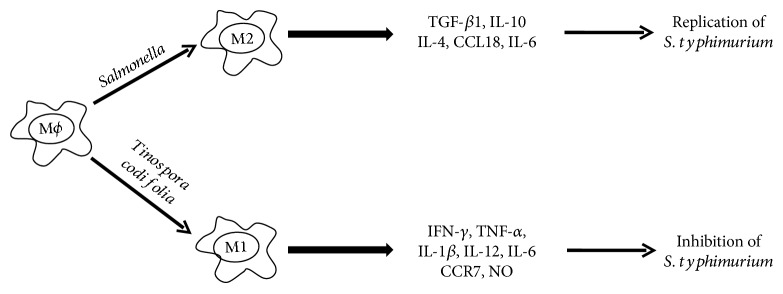
*T. cordifolia* opposes the effect of *S. typhimurium* and polarizes the macrophages towards M1. Treatment of macrophages with *T. cordifolia* results in the secretion of IFN-*γ*, TNF-*α*, and IL-1*β* characteristic of M1 macrophages.

*T. cordifolia* and its constituents have been shown to possess the immune-stimulating properties. *T. cordifolia* and its constituent *α*-D-glucan stimulate NK cells, B cells, and T cells with simultaneous production of various immune-stimulatory cytokines [37, 38]. A polysaccharide from *T. cordifolia*, G1-4A, has been shown to inhibit the intracellular growth of *Mycobacterium tuberculosis* through toll-like receptor 4- (TLR4-) dependent signaling [18]. *Salmonella* spp. use multiple strategies to evade the immune system to establish itself in the host [39]. Macrophages are an important part of the innate immunity and play a critical role in defending the host against the microbial invasion. Classically activated M1 are characterized by the increased secretion of cytokines like TNF-*α*, IL-1*β*, IFN-*γ*, IL-12, and IL-6 and show strong microbicidal activities, whereas alternatively activated macrophages or M2 are characterized by increased secretion of IL-4, IL-10, and TGF-*β* and are considered poorly microbicidal [40, 41]. The results of the present study demonstrated the immune-stimulating activities of AETC and METC as macrophages treated with them secreted higher levels of IL-1*β*, IFN-*γ*, and TNF-*α*. This suggests that *T. cordifolia* extract polarizes the macrophages in the direction of the M1 (Figure 6).

In the present work, we tested the activity of AETC and METC against *S. typhimurium*. *In vitro* and intracellular inhibition of *S. typhimurium* by AETC and METC encouraged us to use them against *S. typhimurium* in a murine model. *Salmonella* spp. manipulate the innate immune signaling to evade the host defense and reside in M2 macrophages [39]. Recognition of *Salmonella* spp. by TLR2 and TL4 is beneficial to the host as mice lacking either or both of these demonstrated increased bacterial burden in the mesenteric lymph nodes [42]. G1-4A, a constituent from *T. cordifolia*, induces TLR-2 signaling that is important to inhibit the intracellular pathogens [18]. This is also supported by the results of the present study that showed *Salmonella*-infected macrophages treated with AETC or METC showed reduced bacterial load as compared to untreated macrophages. Moreover, *S. typhimurium*-infected mice showed increased survival and less bacterial load upon treatment with AETC or METC. METC was found to be more effective as mice treated with METC showed greater survival as compared to those treated with AETC at the same doses. This is in accordance with the results of *in vitro* studies where METC showed greater activity to AETC against *S. typhimurium*. The activity of METC was at par with the standard antibiotic cefixime against murine salmonellosis.

Extensive use of antibiotics causes systemic toxicity and immune-suppression in the treated patients and predisposes them to opportunistic infections. To understand whether the use of AETC or METC is associated with any toxicity, the liver inflammation parameters and the levels of antioxidant enzymes were determined in the blood and tissues of the untreated or treated mice. *S. typhimurium* infection caused liver inflammation as infected mice showed higher levels of ALT and AST in their blood, whereas *Salmonella*-infected mice treated with AETC or METC showed reduced levels of ALT and AST. Thus, *T. cordifolia* did not impart any toxicity, but protected the liver against *S. typhimurium*-induced toxicity. Antioxidant enzymes such as SOD and CAT are an important part of the innate immune response. The levels of SOD and CAT were found to be reduced in *Salmonella*-infected mice, whereas treatment with AETC or METC rescued their levels. These findings support the use of *T. cordifolia* as a hepatoprotective, anti-inflammatory, and antioxidant agent [43, 44].

Immune-stimulating, antimicrobial, anti-inflammatory, and antioxidant activities of *T. cordifolia* may play a substantial therapeutic role against salmonellosis, although more extensive studies are needed before considering *T. cordifolia* as an attractive and safe option in treatment for salmonellosis. Furthermore, this preparation may further be studied for its implications to treat opportunistic infections in immunocompromised persons owing to its immunopotentiating properties.

## 5. Conclusion

In the light of the above results, it can be concluded that aqueous and methanolic extracts (AETC or METC) of *T. cordifolia* possess immune stimulatory, antimicrobial, hepatoprotective, and antioxidant properties. Interestingly, AETC or METC inhibited the intracellular multiplication of *S. typhimurium* in macrophages. Moreover, treatment with AETC or METC was also effective in eliminating *S. typhimurium* infection from infected mice. Therapy with AETC or METC protected the mice against *Salmonella*-induced liver damage and rescued the depleted levels of SOD and CAT in the infected mice. However, further study is needed to explore its potential implication to treat infectious diseases in human population.
